# Analysis of the Nutritional Value of Diets and Food Choices in Polish Female Ulcerative Colitis Individuals Compared with a Pair-Matched Control Sample

**DOI:** 10.3390/nu15040857

**Published:** 2023-02-08

**Authors:** Dominika Głąbska, Dominika Guzek, Gustaw Lech

**Affiliations:** 1 Department of Dietetics, Institute of Human Nutrition Sciences, Warsaw University of Life Sciences (WULS-SGGW), 02-776 Warsaw, Poland; 2 Department of Food Market and Consumer Research, Institute of Human Nutrition Sciences, Warsaw University of Life Sciences (WULS-SGGW), 02-776 Warsaw, Poland; 3 Department of General, Gastroenterological and Oncological Surgery, Medical University of Warsaw, 02-097 Warsaw, Poland

**Keywords:** ulcerative colitis, colitis ulcerous, inflammatory bowel disease, diet, nutritional value, nutrients, food products, intake

## Abstract

Ulcerative colitis patients often attribute their symptoms to specific dietary products. Therefore, even though there are no specific dietary recommendations, these patients commonly have dietary restrictions, often with no consultation from their physician or dietitian, as they believe that they may be beneficial for them. The aim of the study was to analyze the nutritional value of diets and food choices in Polish female ulcerative colitis individuals, in comparison with a pair-matched control sample. The study was conducted on a group of 44 Polish female ulcerative colitis individuals being in remission and 44 individuals within a pair-matched control sample, matched by their age and concurrent diseases, excluding those resulting from ulcerative colitis. The analysis of the diet was based on the self-reported data, including 3-day dietary records (to assess the intake of nutrients and food products), as well as the simple open-ended question about food products excluded from their diet. It was stated that Polish female ulcerative colitis individuals were characterized by a lower energy value of diet (*p* = 0.0043), accompanied by the higher proportion of total protein (*p* = 0.0128) than the pair-matched control sample. As a result of a lower energy value for ulcerative colitis individuals, the intake of numerous nutrients was also lower (*p* < 0.05); however, after recalculation per 1000 kcal, ulcerative colitis individuals were characterized by higher total protein (*p* = 0.0121), starch (*p* = 0.0009), and vitamin B_6_ intake (*p* = 0.0319), as well as lower alcohol intake (*p* = 0.0464). Similarly, as a result of a lower energy value for ulcerative colitis individuals, the intake of numerous foods was also lower (*p* < 0.05); however, after recalculation per 1000 kcal, ulcerative colitis individuals were characterized by higher meat (*p* = 0.0058) and potatoes intake (*p* = 0.0052), as well as lower legumes (*p* = 0.0301), chocolate sweets (*p* = 0.0165), and alcoholic beverages intake (*p* = 0.0062). For chocolate sweets (*p* = 0.0134) and alcoholic beverages (*p* = 0.0091), ulcerative colitis individuals were characterized by a higher frequency of declaration of dietary exclusion. At the same time, ulcerative colitis individuals were characterized by a lower frequency of meeting the recommended intake for magnesium (*p* = 0.0005), iron (*p* = 0.0189), vitamin E (*p* = 0.0389), and vitamin B_1_ (*p* = 0.0032). It was concluded that even in remission, there is a risk of inadequate consumption, not meeting the recommended intake, and nutritional deficiencies in the population of female ulcerative colitis patients.

## 1. Introduction

Inflammatory bowel diseases, including ulcerative colitis and Crohn’s disease, are chronic inflammatory gastrointestinal tract diseases, which result from genetic susceptibility to an exaggerated immune response to a normal stimulus, such as food and intestinal flora [1]. Ulcerative colitis, as the most common inflammatory bowel disease worldwide, is defined as an idiopathic inflammatory colon disease, characterized by diffuse friability and superficial erosions resulting in colonic wall bleeding [2]. 

The prevalence of ulcerative colitis is increasing globally, while the increase is observed mainly in developing countries, which to date, is characterized by a lower ulcerative colitis occurrence, while in developed countries, the incidence is higher, however, this level is relatively stable. Taking this into account, the global yearly incidence varies from 0.5 to 31.5 per 100,000 individuals depending on country [3]. The course of ulcerative colitis is commonly unpredictable, which emerges from alternating periods of remissions and disease exacerbations, while the frequency of exacerbations and their course may differ from mild to fulminant (resulting in the necessity of surgical intervention) [4]. At the same time, the common symptoms of ulcerative colitis, including bloody diarrhea, rectal urgency, tenesmus, and abdominal pain [5] significantly decrease the quality of life of patients [6].

Despite the fact that there are very few well-designed randomized controlled trials assessing the influence of diet on the symptoms and course of ulcerative colitis, patients often attribute their symptoms to specific dietary products [7]. Moreover, despite the evidence-based clinical practice guidelines indicating that there is no need for any special diet therapy for ulcerative colitis patients in remission, these patients commonly have dietary restrictions (which are often not consulted with their physician or dietitian), as they believe that they may be beneficial for them [8]. However, these restrictions and following an improperly balanced diet may contribute to nutritional deficiencies, which is common in ulcerative colitis individuals and results from disease course, complications, applied treatment, and the diet [9].

Despite the fact that there are a limited number of recommendations dedicated for ulcerative colitis patients or inflammatory bowel disease patients, and that it is indicated that the general nutritional recommendations should be followed by these patients while in remission [10], there are some nutritional factors which may be harmful. These observations are commonly made for the western diet [11], meat and processed meat [12], alcoholic beverages [13], and food additives [14]. At the same time, the consumption of fruit, vegetables, fish, soy, olive oil, and fermented dairy as products with anti-inflammatory effects, is indicated as potentially beneficial [15].

The recent Polish study conducted on a population of male ulcerative colitis patients indicated that there were no major differences in the diet between ulcerative colitis patients and the paired-matched sample of healthy individuals, which was stated both for food products and nutrients, but the dietary recommendations were not met in this group [16]. Taking into account the higher risk of deficiencies for ulcerative colitis patients than for healthy individuals [17], it may be a serious problem. Based on the current state of knowledge and considering the important evidence obtained to date for a population of Polish male ulcerative colitis patients, but not for a similar population of female patients, the aim of the study was to analyze the nutritional value of diets and food choices in Polish female ulcerative colitis individuals, in comparison with a pair-matched control sample.

## 2. Materials and Methods

### 2.1. Design of the Study

The study was conducted in accordance with the guidelines of the Declaration of Helsinki, while all the procedures were approved by the Ethical Commission of the Central Clinical Hospital of the Ministry of Interior in Warsaw (registered as 35/2009) and the Ethical Commission of the National Food and Nutrition Institute in Warsaw (registered as 1604/2009). All participants provided their written informed consent for participation in the study. 

The study included an assessment of the nutritional value of diets and food choices in ulcerative colitis individuals, in comparison with a pair-matched control sample. It was conducted by the Dietetic Outpatient Clinic of the Department of Dietetics, Institute of Human Nutrition Sciences, Warsaw University of Life Sciences (WULS-SGGW). The study was conducted within the research project which included the following: (1) Assessment of body composition [18] and diet of ulcerative colitis males [17]; (2) assessment of the influence of potentially beneficial nutrients on the ulcerative colitis symptoms, which was conducted for retinoids [19,20], isoflavone [21,22], and proanthocyaninins [23]; (3) assessment of the influence of diet on the risk of anemia in ulcerative colitis patients [24].

### 2.2. Participants of the Study

The study was conducted on a sample of 44 Polish female ulcerative colitis individuals and a pair-matched sample of 44 healthy individuals. The ulcerative colitis patients were recruited by their gastroenterologists from the following Gastroenterology Outpatient Clinics located in Warsaw (network convenience sampling): Gastroenterology Outpatient Clinic of the Maria Skłodowska-Curie Memorial Cancer Center and Institute of Oncology in Warsaw; Gastroenterology Outpatient Clinic of the Central Clinical Hospital of the Ministry of Interior and Administration in Warsaw; and Gastroenterology Outpatient Clinic of the Public Central Teaching Hospital in Warsaw. Similarly, the control individuals were recruited from the local general medical centers located in Warsaw, as well.

For ulcerative colitis patients, the following inclusion criteria were applied:

− Caucasian;
− Female;
− Age between 18 and 80 years;
− Not hospitalized for any disease at the time;
− Ulcerative colitis diagnosed based on endoscopic examination recorded within clinical records;
− Confirmed clinical remission based on the Mayo Scoring System (the cut-off being 2 points in a 12-points scale) and the Rachmilewitz index (the cut-off being 4 points in a 31-points scale) [25], for at least 6 weeks recorded within clinical records;
− Confirmed endoscopic remission based on an image with no changes or disappearance of the vascular network, erythema, inflammatory polyps allowed;
− Constant drug doses confirmed by the patient for at least 6 weeks.

For ulcerative colitis patients, the following exclusion criteria were applied:

− Pregnancy;
− Cancer diagnosed;
− Condition after any gastrointestinal resection.

For control individuals, the following inclusion criteria were applied:

− Caucasian;
− Female;
− Age between 18 and 80 years;
− Not hospitalized for any disease at the time;
− No diagnosis of ulcerative colitis or any other inflammatory bowel diseases;
− Individually pair-matched with ulcerative colitis patients, matched by their age and concurrent diseases, excluding those resulting from ulcerative colitis and defined as its complications (concurrent diseases clustered based on ICD-10 groups: D50–D89, E00–E07, E10–E16, E78, F00–F99, G00–G99, I10–I15, J00–J99, K00–K46, K65–K93, L00–L99, M00–M99, N99 [26])—the same procedure as for the previous study was applied [17].

For control individuals, the following exclusion criteria were applied:

− Pregnancy;
− Cancer diagnosed;
− Condition after any gastrointestinal resection.

### 2.3. Assessment of the Diet

The analysis of the diet was based on the self-reported data, including the participants’ 3-day dietary records (to assess the intake of nutrients and food products), as well as the simple open-ended question about food products that are excluded from the diet.

The 3-day dietary records were collected using a standard form with detailed instruction to record the consumption of all food products, dishes, and beverages, including the ingredients and preparation techniques of each dish. The participants were asked to conduct their records for 3 random days, but not consecutive ones, with 2 weekdays and 1 weekend day. Each participant was informed about the aim of the study in order to not change their habitual diet for the purpose of conducting the record. The serving sizes were allowed to be expressed in grams (controlled while using a weighing scale or based on information provided by a producer or retailer) or described using standard household measures. If described using standard household measures, each serving size was recalculated into grams by a professional dietitian based on the standard procedure [27]. The 3-day dietary records were provided by 37 respondents from the studied group and 36 respondents from the control group. Thereafter, each 3-day dietary record was recalculated into the energy value of the diet and nutrients intake, as well as the intake of food products, with all of them expressed as the mean daily value.

The energy value of the diet and nutrients intake was calculated using a Polish dietician software Dieta 5.0 (National Food and Nutrition Institute, Warsaw, Poland), developed based on the official tables of the nutritional value of food products [28]. Among the assessed macronutrients, the following were included: total protein (expressed in g and % energy), animal protein (g), plant protein (g), total fat (expressed in g and % energy), saturated fatty acids (SFA) (g), monounsaturated fatty acids (MUFA) (g), polyunsaturated fatty acids (PUFA) (g), cholesterol (mg), total carbohydrates (expressed in g and % energy), sucrose (g), lactose (g), starch (g), fiber (g), and alcohol (expressed in g and % energy). Among the assessed minerals, the following were included: sodium (mg), potassium (mg), calcium (mg), phosphorus (mg), magnesium (mg), iron (mg), zinc (mg), copper (mg), and manganese (mg). Among the assessed vitamins, the following were included: vitamin A (µg retinol equivalents), vitamin E (mg α-tocopherol equivalents), vitamin D (µg), vitamin B_1_ (mg), vitamin B_2_ (mg), niacin (mg), vitamin B_6_ (mg), folate (µg), vitamin B_12_ (µg), and vitamin C (mg). The minerals and vitamins intake was compared with the recommended intake for the age-dependent reference intake values for estimated average requirement (EAR)/adequate intake (AI) values, except for sodium due to the straitened estimation of table salt consumption [29]. As there were differences in the energy value of diet between the ulcerative colitis patients and control group, the intake of each nutrient was additionally recalculated per 1000 kcal of the diet.

The dietary intake was also assessed using DASH index by Mellen et al. [30], as defined by a density-based approach [31]. The included nutrients were as follows: total protein, total fat, SFA, cholesterol, fiber, sodium, potassium, calcium, and magnesium. Moreover, the cut-off recommendations by Mellen et al. [30] were applied and the DASH index of at least 4.5 was interpreted as satisfactory [30].

The food products intake was calculated for the following food groups: milk and dairy beverages (g), cottage cheese (g), rennet cheese (g), eggs (g), meat (g), processed meat products (g), fish and fish products (g), vegetables (g), legumes (g), fruits (g), potatoes (g), bread (g), other cereal products (g), oil (g), margarine (g), butter (g), cream (g), sugar (g), jam and honey (g), chocolate sweets (g), cakes and cookies (g), tea (g), coffee (g), alcoholic beverages (g), sweetened beverages (g), nuts (g), and mushrooms (g). As there were differences in the energy value of diet between the ulcerative colitis patients and control group, the intake of each food group was additionally recalculated per 1000 kcal of the diet.

The question regarding what food products are excluded from the diet was asked as an open-ended question, with a possibility to list an unlimited number of food items or food groups. The respondents were asked to indicate all food items or food groups which they perceive as those that are excluded from their diet. Moreover, they were asked to indicate both food products that were entirely excluded from their diet and those that were only limited, as well as whether the reason for excluding them was associated with perceived gastroenterological symptoms or fear of these symptoms. The information regarding food products excluded from the diet was provided by 34 respondents from the studied group and 37 respondents from the control group. Thereafter, the food products were clustered into food groups as follows: milk and dairy beverages (g), cottage cheese (g), rennet cheese (g), eggs (g), meat (g), processed meat products (g), fish and fish products (g), vegetables (g), legumes (g), fruits (g), potatoes (g), bread (g), other cereal products (g), oil (g), margarine (g), butter (g), cream (g), sugar (g), jam and honey (g), chocolate sweets (g), cakes and cookies (g), tea (g), coffee (g), alcoholic beverages (g), sweetened beverages (g), nuts (g), and mushrooms (g).

### 2.4. Statistical Analysis

The distribution was assessed by the Shapiro-Wilk test. The results were compared using the *t*-Student test (for parametric distributions) and Mann-Whitney U test (for nonparametric distributions). The proportion of individuals in the groups was compared using the chi^2^ test.

The value *p* ≤ 0.05 was interpreted as statistically significant. The statistical analysis was conducted using Statistica 8.0 (StatSoft Inc., Tulsa, OK, USA) and Statgraphics Plus for Windows 5.1 (Statgraphics Technologies Inc., The Plains, VA, USA).

## 3. Results

The macronutrients intake in the studied Polish female ulcerative colitis individuals, in comparison with a pair-matched control sample is presented in Table 1. It was stated that Polish female ulcerative colitis individuals were characterized by a lower energy value of diet (*p* = 0.0043), accompanied by a higher proportion of total protein (*p* = 0.0128) than for a pair-matched control sample. As a result of a lower energy value for ulcerative colitis individuals, the intake of numerous nutrients was also lower, as observed for: plant protein (*p* = 0.0306), total fat (*p* = 0.0063), SFA (*p* = 0.0021), MUFA (*p* = 0.0185), total carbohydrates (*p* = 0.0035), sucrose (*p* = 0.0046), lactose (*p* = 0.0043), fiber (*p* = 0.0039), and alcohol (*p* = 0.0412).

The macronutrients intake recalculated per 1000 kcal in the studied Polish female ulcerative colitis individuals, in comparison with a pair-matched control sample is presented in Table 2. It was stated that Polish female ulcerative colitis individuals were characterized by higher total protein (*p* = 0.0121) and starch intake (*p* = 0.0009), as well as lower alcohol intake (*p* = 0.0464).

The minerals intake in the studied Polish female ulcerative colitis individuals, in comparison with a pair-matched control sample is presented in Table 3. As a result of a lower energy value for ulcerative colitis individuals than for a pair-matched control sample, the intake of numerous minerals was also lower, as observed for: calcium (*p* = 0.0003), phosphorus (*p* = 0.0004), magnesium (*p* < 0.0001), iron (*p* = 0.0023), zinc (*p* = 0.0177), and copper (*p* = 0.0093).

The minerals intake recalculated per 1000 kcal in the studied Polish female ulcerative colitis individuals, in comparison with a pair-matched control sample is presented in Table 4. It was stated that Polish female ulcerative colitis individuals were characterized by comparable minerals intake recalculated per 1000 kcal, in comparison with a pair-matched control sample.

Based on the obtained macronutrients and minerals intake, the DASH index was calculated in the studied Polish female ulcerative colitis individuals, in comparison with a pair-matched control sample. It was stated that the DASH index did not differ between the groups (*p* = 0.6750), amounting to 2.52 ± 1.03 (median of 2.5 differing from 1.0 to 5.0) for ulcerative colitis individuals and 2.69 ± 1.20 (median of 2.5 differing from 1.0 to 6.0) for the control sample. Similarly, the share of individuals meeting the satisfactory DASH index level did not differ (*p* = 0.9748), amounting to 5.5% for ulcerative colitis individuals and 8.1% for the control sample.

The vitamins intake in the studied Polish female ulcerative colitis individuals, in comparison with a pair-matched control sample is presented in Table 5. As a result of a lower energy value for ulcerative colitis individuals than for a pair-matched control sample, the intake of numerous vitamins was also lower, as observed for: vitamin E (*p* = 0.0122), vitamin B_1_ (*p* = 0.0092), vitamin B_2_ (*p* = 0.0043), vitamin B_12_ (*p* = 0.0301), and vitamin C (*p* = 0.0240).

The vitamins intake recalculated per 1000 kcal in the studied Polish female ulcerative colitis individuals, in comparison with a pair-matched control sample is presented in Table 6. It was stated that Polish female ulcerative colitis individuals were characterized by a comparable vitamins intake recalculated per 1000 kcal, as compared with a pair-matched control sample, except for vitamin B_6_, in which ulcerative colitis individuals were characterized by a higher intake (*p* = 0.0319).

The food products intake in the studied Polish female ulcerative colitis individuals, in comparison with a pair-matched control sample is presented in Table 7. As a result of a lower energy value for ulcerative colitis individuals than for a pair-matched control sample, the intake of numerous food products was also lower, as observed for: milk and dairy beverages (*p* = 0.0209), legumes (*p* = 0.0274), oil (*p* = 0.0133), chocolate sweets (*p* = 0.0155), and alcoholic beverages (*p* = 0.0054).

The food products intake recalculated per 1000 kcal in the studied Polish female ulcerative colitis individuals, in comparison with a pair-matched control sample is presented in Table 8. It was stated that Polish female ulcerative colitis individuals were characterized by higher meat (*p* = 0.0058) and potatoes intake (*p* = 0.0052), as well as lower legumes (*p* = 0.0301), chocolate sweets (*p* = 0.0165), and alcoholic beverages intake (*p* = 0.0062).

The proportion of individuals declaring the exclusion of specific food products from their diet in the studied Polish female ulcerative colitis individuals, in comparison with a pair-matched control sample is presented in Table 9. It was stated that Polish female ulcerative colitis individuals were characterized by a comparable frequency of excluding specific food products from their diet in comparison with a pair-matched control sample, except for chocolate sweets (*p* = 0.0134) and alcoholic beverages (*p* = 0.0091), as for them ulcerative colitis individuals were characterized by a higher frequency of declared exclusion from their diet.

The proportion of individuals meeting the recommended intake for minerals and vitamins for the age-dependent reference intake for EAR/AI values in the studied Polish female ulcerative colitis individuals, in comparison with a pair-matched control sample is presented in Table 10. It was stated that Polish female ulcerative colitis individuals were characterized by a lower frequency of meeting the recommended intake for magnesium (*p* = 0.0005), iron (*p* = 0.0189), vitamin E (*p* = 0.0389), and vitamin B_1_ (*p* = 0.0032).

## 4. Discussion

Malnutrition is a common challenge in ulcerative colitis and inflammatory bowel disease patients, since it is often developed during their exacerbations. In this case, the energy expenditure is correlated with the disease activity [32] and may be significantly higher in this period than for healthy individuals [33]. At the same time, consumption during exacerbations must be limited [34]. However, during their remissions, patients commonly tend to limit consumption, as they believe that this approach may allow them to reduce the risk of further exacerbations [35].

This situation was also observed in the presented study, as the Polish female ulcerative colitis individuals were characterized by a lower energy value of diet, accompanied by a lower intake of numerous food products and nutrients, as compared to the pair-matched control sample. However, the value of the diet was similar, as stated for the compared DASH index, which did not differ between the studied groups. Moreover, in comparison with the declared exclusion of food products from their diet, statistically significant differences were stated only for chocolate sweets and alcoholic beverages, as ulcerative colitis individuals were characterized by a higher frequency of declared exclusion from their diet. On the one hand, the lower energy intake may lead to malnutrition. On the other hand, if only chocolate sweets and alcoholic beverages were excluded from the diet more often than for healthy individuals, then it is not the food choice, but the amount of consumed food products that may have been a problem.

As indicated by Casey et al. [36], the effect of ethanol on the gut may be potentially associated with the development of inflammatory bowel disease and impairment of the gut barrier function and permeability [37], along with its proinflammatory actions, including decreasing T-cell activity [38] and increasing TNF-α, interleukin-1, and interleukin-6 levels [39]. At the same time, in ulcerative colitis patients, it was observed that low-to-moderate alcohol drinking compared with heavy drinking was associated with less extensive disease [40]. Finally, the consumption of alcoholic beverages is known to interfere with the metabolism of colitis-specific medications, which may lead to a loss of their therapeutic effect or to an increased risk of side effects [41]. Taking this into account, the declared exclusion of alcoholic beverages must be interpreted as a beneficial behavior in the studied female ulcerative colitis patients.

For chocolate sweets, the situation is not unambiguous, as the chocolate sweets are complex products containing not only cocoa, but also sugar. Therefore, their opposite effects must be considered. In the animal model, the cocoa was observed to inhibit the oxidative effects consequent to colitis, but this effect was not sufficiently strong to remove the intestinal inflammation [42]. At the same time, it was observed to modulate intestinal microbiome and IgA secretion, which consequently modified the functionality of gut-associated lymphoid tissue [43]. However, sugar, similar to the other mono- and disaccharides, is responsible for the increased pro-inflammatory response and reduced gut barrier, which consequently worsens the condition of individuals with colitis [44]. It is associated with some observations made for sugar sweetened beverages, as their consumption in inflammatory bowel disease patients resulted in increased inflammation, disease severity, and frequency of hospitalizations [45]. Despite the potentially positive influence of cocoa, chocolate is a complex product, which should not be attributed to a positive effect for ulcerative colitis patients.

Declared exclusion of chocolate sweets and alcoholic beverages from diet was confirmed by their lower intake in ulcerative colitis patients than in a control group. However, the differences in the consumed amount were also observed for meat and potatoes, as ulcerative colitis patients were characterized by a higher intake, as well as for legumes, as ulcerative colitis patients were characterized by a lower intake for this product. The lower intake of legumes can be undoubtedly interpreted neither as beneficial, nor as harmful, as legumes as a food group contain components of potentially anti-inflammatory properties, including isoflavones, which may relieve ulcerative colitis symptoms [21,22], but also a complex carbohydrate, which is poorly absorbed and as a result, intensifies bacterial fermentation [46]. Similarly as for legumes, the higher intake of potatoes may have its advantages and disadvantages. Whereas glycoalkaloids naturally occurring in potatoes intensified the intestinal inflammation in the animal model of inflammatory bowel disease [47] by affecting intestinal permeability [48], thus the human study revealed that the legumes and potatoes intake was inversely associated with the relapse of inflammatory bowel disease [49]. This may be explained by some antioxidant and anti-inflammatory potential of legumes and potatoes in the intestine [50].

Nevertheless, based on the current state of knowledge, the higher intake of meat observed for ulcerative colitis patients compared with healthy controls must be indicated as a potential problem. The high consumption of meat and processed meat is attributed to a higher risk of ulcerative colitis [51], higher risk of colitis exacerbations [52], and higher mortality in inflammatory bowel disease patients [53]. Moreover, numerous potential mechanisms are herein indicated, including the harmful influence of the sulphide components of meat [12], heterocyclic amines [54], or potential bacterial contamination [55].

The observed differences of food choices between ulcerative colitis patients and healthy individuals influenced the differences of nutrients intake, including the lower intake of alcohol (from alcoholic beverages), as well as the higher intake of protein (from meat products), starch (from potatoes), and vitamin B_6_ (from both meat products and potatoes). In particular, for protein, it must be emphasized that this observation may be of great value, as not only meat products are proven to be potentially harmful for patients in remission, but also protein. The high protein intake is associated with an increased risk of inflammatory bowel disease [56], which may be explained by an influence of protein intake on dysbiosis development [57]. Moreover, similarly as for meat, the high protein intake was observed to be associated with an increased risk of exacerbations in ulcerative colitis [58].

The intake of food products and nutrients is translated into the possibility of meeting the recommended intake. Moreover, the lower intake of numerous food products, associated with the lower energy value of diet of ulcerative colitis patients, as compared with a control group, was associated with a higher risk of inadequate intake. This higher risk of inadequate intake was observed in ulcerative colitis patients for magnesium, iron, vitamin E, and vitamin B_1_, while the indicated nutrients are associated with ulcerative colitis or inflammatory bowel diseases, in terms of the pathophysiology or course of disease. 

For magnesium, the low level of this nutrient is typical for inflammatory bowel diseases [59], which may result from the commonly lower intake than for healthy individuals [60]. Taking into account that in the animal model, magnesium is associated with alleviating colitis [61], it is indicated that it may play a role in preventing exacerbations of inflammatory bowel diseases [62].

For iron deficiency, its crucial consequence, namely iron-deficiency anemia must be indicated, as it is one of the most common manifestations of inflammatory bowel diseases [63]. It results from inadequate dietary intake, which is commonly lower than for healthy individuals [60], but also from malabsorption and chronic blood loss due to mucosal ulcerations [64]. Despite the fact that in general the association between iron and the course of ulcerative colitis itself is not indicated, it is emphasized that recognizing iron-deficiency anemia and correcting the existing deficiency is essential for improving the quality of life and general functioning of inflammatory bowel disease patients [65].

For vitamin E, the systematic review and meta-analysis by Fabisiak et al. [66] has proven the lower status for Crohn’s disease patients than for healthy controls, but within a single study, this situation was also observed for ulcerative colitis patients [67]. It corresponds with the commonly observed inadequate dietary intake [68]. At the same time, in the animal model studies, the beneficial effect of vitamin E was observed, as it even reversed the changes observed during the experimental induction of colitis. Therefore, it was concluded that it may be an effective therapeutic option for ulcerative colitis patients [69].

For vitamin B_1_, the inadequate intake is commonly indicated in female inflammatory bowel disease patients [70]. At the same time, it is stated that this nutrient may be responsible for fatigue in inflammatory bowel disease patients, as its supplementation results in fatigue reduction for this group of patients [71], while a similar effect is observed for other chronic diseases [72].

Taking into account the role of the indicated nutrients for pathophysiology and course of inflammatory bowel diseases, it is necessary to conduct an adequate nutritional education, in order to correct the deficiencies and meet the recommended intake. In particular, for a population of ulcerative colitis female patients, these actions are crucial. For male patients, the intake is similar to the healthy individuals [16], which may suggest more serious restrictions for ulcerative colitis females than for males.

## 5. Conclusions

In this study, it was concluded that in the population of female ulcerative colitis patients, even in remission, there is a risk of inadequate consumption, not meeting the recommended intake, and nutritional deficiencies.

## Figures and Tables

**Table 1:** The macronutrients intake in the studied Polish female ulcerative colitis individuals, in comparison with a pair-matched control sample.

Nutrient—Intake per 24 h	Female Ulcerative Colitis Patients		Pair-Matched Control Sample		p **
	Mean ± SD	Median (Range)	Mean ± SD	Median (Range)	
Energy (kcal)	1724.9 ± 550	1700.0 * (829–3941)	2087.3 ± 497.7	2124.8 (1102.5–3218.9)	0.0043
Total protein (g)	75.5 ± 20.2	76.0 (39.4–115.1)	82.0 ± 16.8	79.8 (57.7–128.6)	0.1374
Total protein (% energy)	18.18 ± 3.64	17.70 (11.39–25.52)	16.50 ± 4.58	16.00 * (9.62–38.40)	0.0128
Animal protein (g)	53.4 ± 18.8	53.9 (10.6–96.0)	57.2 ± 18.1	55.1 (12.4–101.2)	0.3840
Plant protein (g)	21.2 ± 6.5	21.5 (8.4–39.7)	24.8 ± 8.6	25.8 * (4.4–53.3)	0.0306
Total fat (g)	72.6 ± 30.6	66.7 * (30.3–195.2)	90.0 ± 30.2	82.8 * (50.2–194.6)	0.0063
Total fat (% energy)	36.74 ± 6.52	36.14 (20.86–51.02)	37.93 ± 6.11	36.93 (26.82–54.22)	0.4248
Saturated Fatty Acids (SFA) (g)	23.6 ± 11.2	20.8 * (8.1–67.7)	30.8 ± 12.8	26.7 * (15.6–77.9)	0.0021
Monounsaturated Fatty Acids (MUFA) (g)	30.6 ± 14.1	27.0 * (11.8–87.3)	37.8 ± 15.1	34.7 * (9.0–90.5)	0.0185
Polyunsaturated Fatty Acids (PUFA) (g)	12.4 ± 6.4	12.7 * (3.3–26.3)	14.0 ± 6.0	13.5 (3.8–29.2)	0.2104
Cholesterol (mg)	291.7 ± 101.0	299.5 (100.6–586.3)	328.2 ± 130.2	289.9 * (160.6–784.0)	0.3512
Total carbohydrates (g)	206.6 ± 70.4	209.6 * (93.1–450.0)	251.1 ± 74.1	258.6 (51.8–408.0)	0.0035
Total carbohydrates (% energy)	44.82 ± 7.39	46.65 * (22.20–63.63)	44.23 ± 8.26	47.06 * (16.03–58.89)	0.9560
Sucrose (g)	40.5 ± 26.1	39.3 * (2.9–118.3)	57.3 ± 28.3	57.6 (5.8–120.5)	0.0046
Lactose (g)	7.7 ± 6.6	5.6 * (0.0–29.6)	13.0 ± 10.6	9.9 * (2.0–53.2)	0.0072
Starch (g)	116.1 ± 41.6	117.1 (49.8–238.1)	117.2 ± 44.0	125.5 (9.1–211.4)	0.9151
Fiber (g)	16.3 ± 5.4	16.4 (4.6–30.8)	21.5 ± 8.4	20.2 * (8.4–51.4)	0.0039
Alcohol (g)	0.7 ± 2.3	0.0 * (0.0–9.6)	3.8 ± 7.2	0.0 * (0.0–32.7)	0.0412
Alcohol (% energy)	0.26 ± 0.90	0.00 * (0.00–3.72)	1.25 ± 2.68	0.00 * (0.00–12.03)	0.0730

* Nonparametric distribution (Shapiro-Wilk test—*p* ≤ 0.05); ** compared using the *t*-Student test (for parametric distributions)/Mann-Whitney U test (for nonparametric distributions).

**Table 2:** The macronutrients intake recalculated per 1000 kcal in the studied Polish female ulcerative colitis individuals, in comparison with a pair-matched control sample.

Nutrient—Recalculated per 1000 kcal	Female Ulcerative Colitis Patients		Pair-Matched Control Sample		p **
	Mean ± SD	Median (Range)	Mean ± SD	Median (Range)	
Total protein (g)	44.9 ± 9.0	43.9 (28.2–63.2)	40.7 ± 11.4	39.5 * (24.1–95.8)	0.0121
Animal protein (g)	31.9 ± 10.3	30.2 (7.1–51.1)	28.9 ± 13.2	27.2 * (4.6–91.8)	0.0730
Plant protein (g)	12.5 ± 2.1	12.9 (8.0–17.3)	11.9 ± 3.1	11.0 * (4.0–19.5)	0.1800
Total fat (g)	41.5 ± 7.4	40.9 (23.4–57.7)	42.8 ± 6.9	41.6 (30.5–61.3)	0.4198
Saturated Fatty Acids (SFA) (g)	13.4 ± 3.3	13.2 (7.4–22.8)	14.7 ± 3.8	14.7 * (9.2–28.5)	0.1628
Monounsaturated Fatty Acids (MUFA) (g)	17.5 ± 4.1	17.0 (7.6–25.7)	14.8 ± 4.6	17.1 (6.2–29.7)	0.7479
Polyunsaturated Fatty Acids (PUFA) (g)	7.1 ± 2.8	6.6 (2.1–14.0)	6.6 ± 2.1	6.9 (2.4–13.3)	0.4426
Cholesterol (mg)	174.6 ± 56.88	171.6 * (63.7–366.5)	160.5 ± 70.2	145.1 * (76.6–491.9)	0.0748
Total carbohydrates (g)	120.4 ± 19.6	125.1 * (60.5–169.9)	119.3 ± 21.3	125.1 * (47.0–155.4)	0.9956
Sucrose (g)	23.0 ± 11.4	24.4 (3.5–48.6)	26.7 ± 11.1	24.7 (5.2–46.9)	0.1652
Lactose (g)	4.5 ± 3.8	3.5 (0.0–19.8)	6.3 ± 4.4	5.0 * (0.7–16.5)	0.0502
Starch (g)	67.5 ± 13.9	64.5 (39.6–89.7)	55.1 ± 16.6	56.0 (8.3–92.8)	0.0009
Fiber (g)	9.8 ± 3.0	9.9 (4.3–15.6)	10.3 ± 3.2	9.4 * (5.5–18.8)	0.7199
Alcohol (g)	0.4 ± 1.3	0.0 * (0.0–5.4)	1.9 ± 3.9	0.0 * (0.0–17.3)	0.0464

* Nonparametric distribution (Shapiro-Wilk test—*p* ≤ 0.05); ** compared using the *t*-Student test (for parametric distributions)/Mann-Whitney U test (for nonparametric distributions).

**Table 3:** The minerals intake in the studied Polish female ulcerative colitis individuals, in comparison with a pair-matched control sample.

Nutrient—Intake per 24 h	Female Ulcerative Colitis Patients		Pair-Matched Control Sample		p **
	Mean ± SD	Median (Range)	Mean ± SD	Median (Range)	
Sodium (mg)	1770.7 ± 643.5	1827.2 * (938.5–3346.6)	1956.2 ± 544.9	1857.0 * (1186.4–3550.2)	0.1159
Potassium (mg)	3087.4 ± 796.0	3208.3 (976.5–4437.8)	3554.5 ± 1038.8	3139.5 * (2070.6–6101.1)	0.2065
Calcium (mg)	535.5 ± 238.1	495.0 * (205.1–1417.5)	762.1 ± 307.2	732.7 * (400.1–1963.2)	0.0003
Phosphorus (mg)	1111.6 ± 297.9	1089.7 (569.0–1806.8)	1380.1 ± 285.6	1364.8 * (992.8–2345.8)	0.0004
Magnesium (mg)	257.0 ± 69.6	251.2 * (88.8–405.0)	339.4 ± 88.3	331.6 (171.1–526.3)	<0.0001
Iron (mg)	10.05 ± 3.06	9.67 * (4.15–17.35)	12.48 ± 3.39	12.19 (6.12–21.14)	0.0023
Zinc (mg)	9.28 ± 2.82	9.22 * (4.70–16.90)	10.93 ± 2.82	10.40 (6.36–19.06)	0.0177
Copper (mg)	1.06 ± 0.35	1.02 * (0.29–1.76)	1.31 ± 0.41	1.24 (0.64–2.58)	0.0093
Manganese (mg)	4.91 ± 1.89	4.91 * (0.75–10.88)	6.00 ± 2.81	5.19 (1.51–11.72)	0.1800

* Nonparametric distribution (Shapiro-Wilk test—*p* ≤ 0.05); ** compared using the *t*-Student test (for parametric distributions)/Mann-Whitney U test (for nonparametric distributions).

**Table 4:** The minerals intake recalculated per 1000 kcal in the studied Polish female ulcerative colitis individuals, in comparison with a pair-matched control sample.

Nutrient—Intake per 1000 kcal	Female Ulcerative Colitis Patients		Pair-Matched Control Sample		p **
	Mean ± SD	Median (Range)	Mean ± SD	Median (Range)	
Sodium (mg)	1051.8 ± 299.7	1045.4 (502.8–1838.9)	965.9 ± 256.1	974.5 (434.0–1545.5)	0.1926
Potassium (mg)	1853.2 ± 479.8	1854.7 (928.1–3045.5)	1713.6 ± 320.9	1671.3 (1217.0–2533.0)	0.1496
Calcium (mg)	322.5 ± 135.9	300.9 * (104.6–814.4)	373.7 ± 128.3	353.7 (142.6–675.5)	0.0528
Phosphorus (mg)	662.5 ± 135.0	658.3 (363.9–981.8)	682.8 ± 168.9	684.9 * (455.9–1473.2)	0.5926
Magnesium (mg)	152.0 ± 34.2	144.7 (84.4–223.8)	165.8 ± 36.9	159.0 (103.9–266.9)	0.1034
Iron (mg)	5.96 ±1.35	5.61 (3.94–9.35)	6.00 ± 1.12	6.20 (3.62–8.19)	0.8056
Zinc (mg)	5.50 ± 1.17	5.42 (3.28–8.52)	5.30 ± 0.97	5.26 (3.82–7.56)	0.4486
Copper (mg)	0.63 ± 0.16	0.63 (0.28–0.93)	0.63 ± 0.14	0.59 (0.40–0.94)	0.8681
Manganese (mg)	3.00 ± 1.35	3.00 (0.71–6.36)	2.89 ± 1.31	2.73 * (1.09–6.94)	0.5624

* Nonparametric distribution (Shapiro-Wilk test—*p* ≤ 0.05); ** compared using the *t*-Student test (for parametric distributions)/Mann-Whitney U test (for nonparametric distributions).

**Table 5:** The vitamins intake in the studied Polish female ulcerative colitis individuals, in comparison with a pair-matched control sample.

Nutrient—Intake per 24 h	Female Ulcerative Colitis Patients		Pair-Matched Control Sample		p **
	Mean ± SD	Median (Range)	Mean ± SD	Median (Range)	
Vitamin A (µg retinol equivalents)	1180.1 ± 1468.7	906.3 * (257.3–9498.0)	1417.3 ± 939.0	1137.7 * (284.9–4026.0)	0.0528
Vitamin E (mg α-tocopherol equivalents)	10.84 ± 4.92	10.83 * (3.36–25.10)	14.30 ± 6.60	13.66 * (2.67–31.13)	0.0122
Vitamin D (µg)	3.72 ± 3.89	2.48 * (0.97–18.54)	4.04 ± 3.64	2.70 * (0.61–16.51)	0.7240
Vitamin B_1_ (mg)	1.48 ± 2.35	0.99 * (0.59–15.04)	1.41 ± 0.63	1.29 * (0.65–3.84)	0.0092
Vitamin B_2_ (mg)	1.53 ± 0.55	1.47 * (0.74–3.84)	1.81 ± 0.45	1.69 * (1.19–3.51)	0.0043
Niacin (mg)	16.69 ± 5.40	16.04 (4.44–31.35)	19.09 ± 7.39	17.79 * (8.47–44.47)	0.2820
Vitamin B_6_ (mg)	1.92 ± 0.49	1.86 (0.73–2.92)	2.16 ± 0.69	2.05 (1.06–3.84)	0.1016
Folate (µg)	301.3 ± 92.7	298.5 (91.3–507.8)	356.3 ± 135.7	319.6 * (153.1–759.4)	0.1407
Vitamin B_12_ (µg)	5.59 ± 10.93	3.46 * (1.06–68.48)	4.57 ± 1.94	4.11 * (1.56–10.75)	0.0301
Vitamin C (mg)	101.3 ± 49.6	95.3 (8.4–200.5)	149.0 ± 98.8	128.9 * (6.7–540.5)	0.0240

* Nonparametric distribution (Shapiro-Wilk test—*p* ≤ 0.05); ** compared using the *t*-Student test (for parametric distributions)/Mann-Whitney U test (for nonparametric distributions).

**Table 6:** The vitamins intake recalculated per 1000 kcal in the studied Polish female ulcerative colitis individuals, in comparison with a pair-matched control sample.

Nutrient—Intake per 1000 kcal	Female Ulcerative Colitis Patients		Pair-Matched Control Sample		p **
	Mean ± SD	Median (Range)	Mean ± SD	Median (Range)	
Vitamin A (µg retinol equivalents)	685.1 ± 787.8	553.5 * (219.4–5161.3)	669.1 ± 378.2	597.2 * (204.9–1786.8)	0.5330
Vitamin E (mg α-tocopherol equivalents)	6.29 ± 2.11	6.24 (3.19–11.33)	6.73 ± 2.55	6.31 * (2.42–15.72)	0.4767
Vitamin D (µg)	2.2 ± 2.1	1.4 * (0.5–10.3)	1.94 ± 1.87	1.37 * (0.36–10.10)	0.4432
Vitamin B_1_ (mg)	0.86 ± 1.26	0.61 * (0.37–8.17)	0.67 ± 0.21	0.66 * (0.37–1.51)	0.6550
Vitamin B_2_ (mg)	0.91 ± 0.27	0.88 * (0.49–2.09)	0.88 ± 0.19	0.86 * (0.55–1.54)	0.7954
Niacin (mg)	10.06 ± 3.43	9.33 (5.08–20.09)	9.52 ± 5.60	8.66 * (5.91–40.34)	0.1320
Vitamin B_6_ (mg)	1.16 ± 0.28	1.16 (0.64–1.81)	1.04 ± 0.26	0.95 * (0.72–2.02)	0.0319
Folate (µg)	182.2 ± 58.8	171.6 * (86.8–369.3)	171.7 ± 51.3	154.2 * (110.3–304.1)	0.4111
Vitamin B_12_ (µg)	3.2 ± 5.9	2.0 * (0.7–37.2)	2.26 ± 0.96	2.01 * (0.57–4.90)	0.7117
Vitamin C (mg)	63.9 ± 40.7	55.2 * (7.1–185.1)	71.7 ±43.9	60.7 * (6.1–242.2)	0.3744

* Nonparametric distribution (Shapiro-Wilk test—*p* ≤ 0.05); ** compared using the *t*-Student test (for parametric distributions)/Mann-Whitney U test (for nonparametric distributions).

**Table 7:** The food products intake in the studied Polish female ulcerative colitis individuals, in comparison with a pair-matched control sample.

Food Products—Intake per 24 h	Female Ulcerative Colitis Patients		Pair-Matched Control Sample		p **
	Mean ± SD	Median (Range)	Mean ± SD	Median (Range)	
Milk and dairy beverages (g)	105.1 ± 126.6	62.0 * (0.0–585.0)	197.0 ± 212.6	113.5 * (0.0–1057.0)	0.0209
Cottage cheese (g)	40.0 ± 42.7	20.0 * (0.0–160.0)	45.1 ± 63.2	21.5 * (0.0–325.0)	0.9240
Rennet cheese (g)	16.7 ± 28.1	5.0 * (0.0–150.0)	20.1 ± 22.3	13.0 * (0.0–73.0)	0.2362
Eggs (g)	23.0 ± 21.2	20.0 * (0.0–70.0)	23.4 ± 21.9	17.5 * (0.0–86.0)	0.8769
Meat (g)	142.1 ± 83.8	136.2 * (0.0–415.0)	120.3 ± 89.3	121.2 * (0.0–353.7)	0.1209
Processed meat products (g)	37.9 ± 37.5	35.0 * (0.0–140.0)	42.7 ± 41.0	33.5 * (0.0–187.0)	0.6395
Fish and fish products (g)	33.3 ± 47.9	0.0 * (0.0–167.0)	28.0 ± 35.1	21.0 * (0.0–133.0)	0.9115
Vegetables (g)	226.3 ± 145.5	201.2 (0.0–640.0)	292.5 ± 170.8	236.5 * (24.7–816.5)	0.1096
Legumes (g)	0.1 ± 0.4	0.0 * (0.0–2.0)	1.9 ± 4.1	0.0 * (0.0–15.0)	0.0274
Fruits (g)	153.6 ± 138.2	125.9 * (0.0–494.1)	233.7 ± 205.5	182.9 * (0.0–1083.5)	0.0975
Potatoes (g)	134.7 ± 91.6	134.0 (0.0–383.0)	104.1 ± 102.7	83.0 * (0.0–428.0)	0.1017
Bread (g)	92.2 ± 66.1	78.0 * (0.0–260.0)	88.8 ± 57.7	74.0 * (0.0–292.0)	0.9209
Other cereal products (g)	49.5 ± 58.8	36.0 * (0.0–254.0)	60.4 ± 46.6	43.5 * (3.0–185.0)	0.0729
Oil (g)	13.6 ± 12.8	11.0 * (0.0–51.0)	23.2 ± 17.1	25.0 (0.0–73.0)	0.0133
Margarine (g)	6.9 ± 9.6	3.0 * (0.0–42.0)	5.7 ± 10.7	1.0 * (0.0–42.0)	0.1701
Butter (g)	7.5 ± 10.8	4.0 * (0.0–51.0)	11.1 ± 12.3	9.0 * (0.0–64.0)	0.0551
Cream (g)	5.7 ± 8.9	0.0 * (0.0–34.0)	6.7 ± 10.1	0.5 * (0.0–50.0)	0.7093
Sugar (g)	7.5 ± 12.7	1.0 * (0.0–47.0)	8.3 ± 12.0	1.0 * (0.0–39.0)	0.9819
Jam and honey (g)	8.5 ± 12.1	0.0 * (0.0–40.0)	11.7 ± 17.2	0.0 * (0.0–55.0)	0.5551
Chocolate sweets (g)	12.8 ± 27.5	0.0 * (0.0–146.0)	24.1 ± 34.0	13.5 * (0.0–178.0)	0.0155
Cakes and cookies (g)	41.6 ± 53.5	17.0 * (0.0–200.0)	46.1 ± 61.9	26.0 * (0.0–253.0)	0.7001
Tea (g)	602.2 ± 422.1	660.0 (0.0–1417.0)	607.0 ± 473.6	521.0 * (0.0–1667.0)	0.7993
Coffee (g)	171.9 ± 132.7	170.0 * (0.0–417.0)	179.9 ± 161.6	158.5 * (0.0–500.0)	0.8805
Alcoholic beverages (g)	7.2 ± 25.0	0.0 * (0.0–100.0)	49.1 ± 106.4	0.0 * (0.0–477.0)	0.0054
Sweetened beverages (g)	60.2 ± 124.2	0.0 * (0.0–533.0)	82.2 ± 154.2	0.0 * (0.0–610.0)	0.4052
Nuts (g)	6.5 ± 17.1	0.0 * (0.0–89.3)	6.1 ± 14.2	0.0 * (0.0–70.7)	0.8173
Mushrooms (g)	2.5 ± 9.9	0.0 * (0.0–56.0)	2.4 ± 6.6	0.0 * (0.0–31.1)	0.5104

* Nonparametric distribution (Shapiro-Wilk test—*p* ≤ 0.05); ** compared using the *t*-Student test (for parametric distributions)/Mann-Whitney U test (for nonparametric distributions).

**Table 8:** The food products intake recalculated per 1000 kcal in the studied Polish female ulcerative colitis individuals, in comparison with a pair-matched control sample.

Food Products—Intake per 1000 kcal	Female Ulcerative Colitis Patients		Pair-Matched Control Sample		p **
	Mean ± SD	Median (Range)	Mean ± SD	Median (Range)	
Milk and dairy beverages (g)	60.9 ± 74.7	42.0 * (0.0–392.6)	97.2 ± 60.1	66.8 * (0.0–328.4)	0.0566
Cottage cheese (g)	24.0 ± 25.6	14.3 * (0.0–85.3)	25.1 ± 45.2	8.5 * (0.0–252.3)	0.4293
Rennet cheese (g)	10.1 ± 17.0	2.3 * (0.0–86.2)	9.9 ± 11.6	6.2 * (0.0–38.9)	0.3572
Eggs (g)	14.7 ± 16.9	9.4 * (0.0–80.8)	11.7 ± 12.0	7.8 * (0.0–60.8)	0.6384
Meat (g)	84.2 ± 47.7	84.2 * (0.0–252.4)	61.8 ± 57.1	53.0 * (0.0–320.9)	0.0058
Processed meat products (g)	23.0 ± 21.7	17.7 * (0.0–74.2)	23.0 ± 29.7	16.3 * (0.0–169.6)	0.6759
Fish and fish products (g)	18.6 ± 26.8	0.0 * (0.0–108.1)	13.6 ± 18.7	7.1 * (0.0–81.3)	0.8653
Vegetables (g)	137.7 ± 93.6	111.8 * (0.0–412.3)	139.5 ± 79.0	123.5 * (22.4–383.6)	0.6871
Legumes (g)	0.1 ± 0.4	0.0 * (0.0–2.2)	1.0 ± 2.0	0.0 * (0.0–6.7)	0.0301
Fruits (g)	100.4 ± 105.8	66.7 * (0.0–419.9)	107.1 ± 92.1	85.7 * (0.0–485.5)	0.3449
Potatoes (g)	80.9 ± 57.0	68.3 (0.0–230.5)	45.8 ± 44.4	44.7 * (0.0–214.4)	0.0052
Bread (g)	53.3 ± 33.7	45.6 (0.0–124.2)	41.9 ± 28.7	40.0 * (0.0–155.1)	0.1800
Other cereal products (g)	27.8 ± 29.5	21.9 * (0.0–124.8)	28.1 ± 21.1	20.5 * (1.9–84.8)	0.6549
Oil (g)	7.7 ± 6.6	7.1 * (0.0–22.8)	11.5 ± 9.0	9.4 * (0.0–42.6)	0.0783
Margarine (g)	4.2 ± 5.5	1.4 * (0.0–23.2)	2.4 ± 4.4	0.5 * (0.0–17.0)	0.0709
Butter (g)	3.9 ± 4.6	3.3 * (0.0–23.6)	4.9 ± 4.6	4.6 * (0.0–23.4)	0.2283
Cream (g)	3.3 ± 5.1	0.0 * (0.0–17.2)	3.3 ± 4.7	0.4 * (0.0–18.3)	0.8918
Sugar (g)	4.1 ± 6.5	1.0 * (0.0–25.5)	4.1 ± 6.0	0.6 * (0.0–21.3)	0.8076
Jam and honey (g)	4.6 ± 6.7	0.0 * (0.0–24.6)	6.4 ± 10.0	0.0 * (0.0–34.6)	0.6659
Chocolate sweets (g)	6.5 ± 13.1	0.0 * (0.0–65.6)	10.7 ± 13.7	5.6 * (0.0–67.7)	0.0165
Cakes and cookies (g)	24.1 ± 30.5	12.0 * (0.0–118.7)	22.9 ± 29.6	10.6 * (0.0–113.0)	0.9503
Tea (g)	387.5 ± 340.5	322.2 * (0.0–1375.3)	260.1 ± 184.5	244.7 * (0.0–610.9)	0.2741
Coffee (g)	104.2 ± 83.6	99.6 * (0.0–307.0)	97.2 ± 92.1	99.6 * (0.0–346.3)	0.6521
Alcoholic beverages (g)	4.0 ± 13.9	0.0 * (0.0–59.3)	26.6 ± 58.8	0.0 * (0.0–254.1)	0.0062
Sweetened beverages (g)	30.3 ± 62.7	0.0 * (0.0–254.2)	40.8 ± 75.3	0.0 * (0.0–301.1)	0.3878
Nuts (g)	3.4 ± 8.2	0.0 * (0.0–41.7)	3.3 ± 7.7	0.0 * (0.0–37.6)	0.8348
Mushrooms (g)	1.3 ± 5.0	0.0 * (0.0–28.3)	1.0 ± 2.6	0.0 * (0.0–9.8)	0.5553

* Nonparametric distribution (Shapiro-Wilk test—*p* ≤ 0.05); ** compared using the *t*-Student test (for parametric distributions)/Mann-Whitney U test (for nonparametric distributions).

**Table 9:** The proportion of individuals declaring the exclusion of specific food products from their diet in the studied Polish female ulcerative colitis individuals, in comparison with a pair-matched control sample.

Food Products	Female Ulcerative Colitis Patients	Pair-Matched Control Sample	p *
Milk and dairy beverages	21.6	8.3	0.2079
Cottage cheese	29.7	33.3	0.9367
Rennet cheese	48.6	33.3	0.2749
Eggs	21.6	11.1	0.3705
Meat	8.1	8.3	1.0000
Processed meat products	32.4	22.2	0.4743
Fish and fish products	51.4	44.4	0.7216
Vegetables	5.4	0.0	0.4855
Legumes	94.6	77.8	0.0803
Fruits	16.2	11.1	0.7689
Potatoes	10.8	22.2	0.3176
Bread	8.1	5.6	1.0000
Other cereal products	10.8	0.0	0.1298
Oil	24.3	13.9	0.4038
Margarine	29.7	44.4	0.2894
Butter	29.7	16.7	0.2968
Cream	51.4	50.0	1.0000
Sugar	35.1	38.9	0.9285
Jam and honey	59.5	52.8	0.7344
Chocolate sweets	62.2	30.6	0.0134
Cakes and cookies	40.5	33.3	0.6927
Tea	13.5	13.9	1.0000
Coffee	24.3	27.8	0.9446
Alcoholic beverages	91.9	63.9	0.0091
Sweetened beverages	67.6	55.6	0.4154
Nut	78.4	75.0	0.9486
Mushrooms	86.5	80.6	0.7131

* Compared using the chi^2^ test.

**Table 10:** The proportion of individuals meeting the recommended intake for minerals and vitamins for the age-dependent reference intake values for estimated average requirement (EAR)/adequate intake (AI) values in the studied Polish female ulcerative colitis individuals, in comparison with a pair-matched control sample.

Nutrients		Female Ulcerative Colitis Patients		Pair-Matched Control Sample		p *
		Intake Lower Than Recommended	Recommended Intake Met	Intake Lower Than Recommended	Recommended Intake Met	
Minerals	Potassium	100.0	0.0	88.9	11.1	0.1161
	Calcium	97.3	2.7	83.3	16.7	0.1035
	Phosphorus	5.4	94.6	0.0	100.0	0.4855
	Magnesium	62.2	37.8	19.4	80.6	0.0005
	Iron	18.9	81.1	0.0	100.0	0.0189
	Zinc	18.9	81.1	2.8	97.2	0.0669
	Copper	10.8	89.2	2.8	97.2	0.3707
Vitamins	Vitamin A	13.5	86.5	8.3	91.7	0.7386
	Vitamin E	37.8	62.2	13.9	86.1	0.0389
	Vitamin D	89.2	10.8	77.8	22.2	0.3176
	Vitamin B_1_	48.6	51.4	13.9	86.1	0.0032
	Vitamin B_2_	8.1	91.9	0.0	100.0	0.2481
	Niacin	8.1	91.9	8.3	91.7	1.0000
	Vitamin B_6_	8.1	91.9	5.6	94.4	1.0000
	Folate	64.9	35.1	47.2	52.8	0.1995
	Vitamin B_12_	16.2	83.8	2.8	97.2	0.1206
	Vitamin C	18.9	81.1	13.9	86.1	0.7919

* Compared using the chi^2^ test.

## Data Availability

The data presented in this study are available on request from the corresponding author.
